# Research on Key Factors and Their Interaction Effects of Electromagnetic Force of High-Speed Solenoid Valve

**DOI:** 10.1155/2014/567242

**Published:** 2014-08-26

**Authors:** Peng Liu, Liyun Fan, Qaisar Hayat, De Xu, Xiuzhen Ma, Enzhe Song

**Affiliations:** College of Power and Energy Engineering, Harbin Engineering University, Harbin 150001, China

## Abstract

Analysis consisting of numerical simulations along with lab experiments of interaction effects between key parameters on the electromagnetic force based on response surface methodology (RSM) has been also proposed to optimize the design of high-speed solenoid valve (HSV) and improve its performance. Numerical simulation model of HSV has been developed in Ansoft Maxwell environment and its accuracy has been validated through lab experiments. Effect of change of core structure, coil structure, armature structure, working air gap, and drive current on the electromagnetic force of HSV has been analyzed through simulation model and influence rules of various parameters on the electromagnetic force have been established. The response surface model of the electromagnetic force has been utilized to analyze the interaction effect between major parameters. It has been concluded that six interaction factors including working air gap with armature radius, drive current with armature thickness, coil turns with side pole radius, armature thickness with its radius, armature thickness with side pole radius, and armature radius with side pole radius have significant influence on the electromagnetic force. Optimal match values between coil turns and side pole radius; armature thickness and side pole radius; and armature radius and side pole radius have also been determined.

## 1. Introduction

HSV is one of the most critical components of electronic control fuel system (ECFS) whose strong electromagnetic force and rapid response characteristics have a great influence on the flexible fuel injection of ECFS [1–4]. Need for a high-speed performance solenoid valve has been increased in order to meet the increasingly stringent emission regulations and improve fuel economy. Therefore it is of great significance to carry out research work on HSV.

Even though there are lots of research reports on the subject, most of them focus on the modeling, dynamic response experiment, and control method of HSV. In [5], different finite-element approaches for electromechanical dynamics were presented and compared. In [6], a simulation model of solenoid value which included a mechanical submodel and a magnetic submodel was developed and validated. In [7, 8], a comprehensive multiphysics theoretical model of a solenoid valve was constructed using the finite-element method, and it could provide useful information on the temperature distribution, mechanical and thermal deformations, and stresses. In [9, 10], a test equipment of dynamic response characteristics for solenoid valve was designed and the influence of different parameters on dynamic response were studied through experiment. In [11], a new kind of driving module of solenoid value was presented, and it could shorten solenoid responding time, reduce energy consumption, and decrease software complexity. In [12], the method of indirect adaptive closed loop control was described; this method could enable robust detection and control of closure time and hold current. In [13], the impact of different control strategies applied to driving the solenoid injector was investigated and a high correlation was found between the opening delay and the solenoid current. The switch time could be further optimized through the proposed driving circuits and control strategies.

The solenoid value itself must be studied in an early stage of system development in order to develop a comparative HSV system. The research on electromagnetic force key parameters of HSV is of great significance for designing and optimizing HSV, but it has been rarely reported. In addition, interaction effect between these different parameters and their combined effect on the electromagnetic force also require further research. Therefore in this paper, numerical simulations have been carried out along with lab experiments and analysis method based on RSM has been proposed to investigate the interaction effects between key parameters on the electromagnetic force of HSV. Effect of individual parameters of HSV have been thoroughly analyzed which is followed by further investigations considering the interaction effect between two parameters. Response surface model of electromagnetic force has been obtained and significant interaction factors have been analyzed. Research work carried out in this paper decisively provides certain theoretical guidance for the design and optimization of HSV.

**Figure 1:**
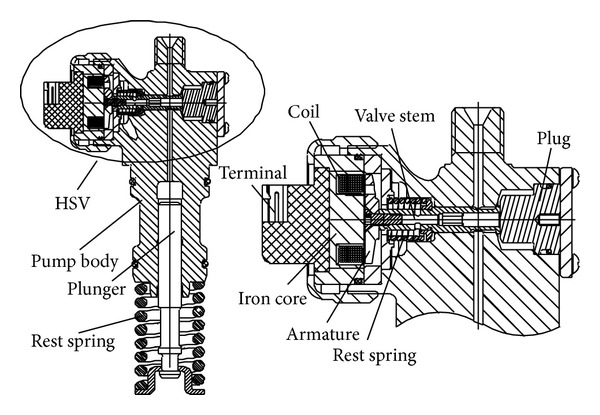
Schematic of EUP and HSV.

This paper focuses on HSV of electronic unit pump (EUP) shown in Figure 1. EUP mainly includes HSV, pump body, plunger, and its rest spring. HSV mainly includes armature, iron core, coil, valve stem, reset spring, terminal, and plug. After turning the power on, iron core attracts armature; pulls the valve stem; closes the seal cone; cuts off fuel loop; and thus sets up the high pressure in the pump chamber which is required for fuel injection. Whereas turning power off resets all. Reset spring forces armature to reset valve stem, decreasing the high pressure fuel inside pump chamber and stopping the fuel injection. Controlling of injection timing and injection quantity can be achieved through precisely adjusting the closing time and duration of control valve stem [14].

## 2. Methodology

In this paper we have carried out our research work from the perspective of HSV magnetostatic characteristics which mainly refers to the electromagnetic force characteristics on the condition that mechanical system and electromagnetic system of HSV are both in steady state. All sorts of transition states need not be considered for the computation of electromagnetic force. The electromagnetic force is determined by the steady current of the coil and the design of HSV itself.

**Figure 2:**
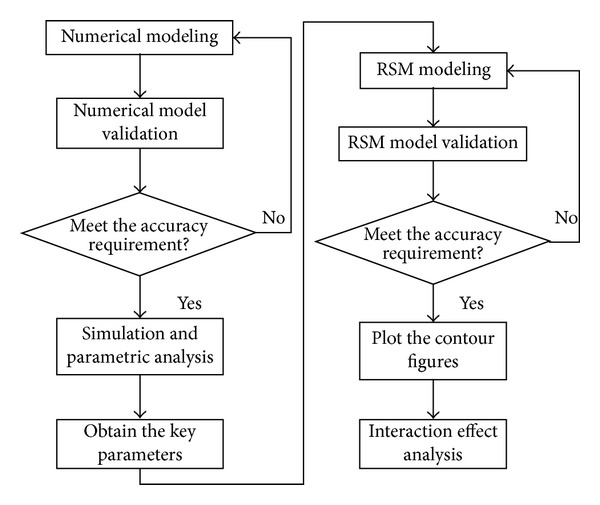
Research flowchart.

Figure 2 shows the entire research method and process. Firstly, numerical model of HSV was developed and validated. Secondly, electromagnetic characteristics parameters were analyzed using the numerical model and key influencing parameters were obtained. Thirdly, response surface model of the key parameters was developed and verified. And finally, interaction effect was analyzed by drawing contour map.

### 2.1. Numerical Modeling

#### 2.1.1. Electromagnetic Force Computation Theory

Numerical simulation model of HSV has been developed in Ansoft Maxwell environment. The virtual work method is employed to compute the electromagnetic force in Ansoft Maxwell software. Armature is virtually displaced by “*s*” and the electromagnetic force on the armature in the direction of the displacement “*s*” is given by the following relationship:

(1) $$\begin{array}{c} F={\frac{\text{d}W\left(s,i\right)}{\text{d}s}|}_{i=\text{constant}}. \end{array}$$

In the above equation *W*(*s*, *i*) is the magnetic coenergy of the system and *i* is the steady current of coil. *W*(*s*, *i*) is given by

(2) $$\begin{array}{c} W\left(s,i\right)=\overset{}{\underset{V}{\int }}\left(\overset{H}{\underset{0}{\int }}B\cdot \text{d}H\right)\text{d}V, \end{array}$$

where *V* is the virtually distorted empty area around the armature, *H* is the magnetic field, and *B* is the magnetic induction intensity.

After combining (1) with (2), we get

(3) $$\begin{array}{c} F=\frac{\partial }{\partial s}\left[\overset{}{\underset{V}{\int }}\left(\overset{H}{\underset{0}{\int }}B\cdot \text{d}H\right)\text{d}V\right]. \end{array}$$

Maxwell equations for above magnetostatic field can be simplified as

(4) $$\begin{array}{c} \nabla \times H=J,\\ \nabla \cdot B=0. \end{array}$$

The finite-element method is employed to solve (4) and then *H* and *B* of entire solution domain are obtained. Finally, the electromagnetic force in (3) is calculated.

#### 2.1.2. Numerical Modeling in Ansoft Maxwell

**Figure 3:**
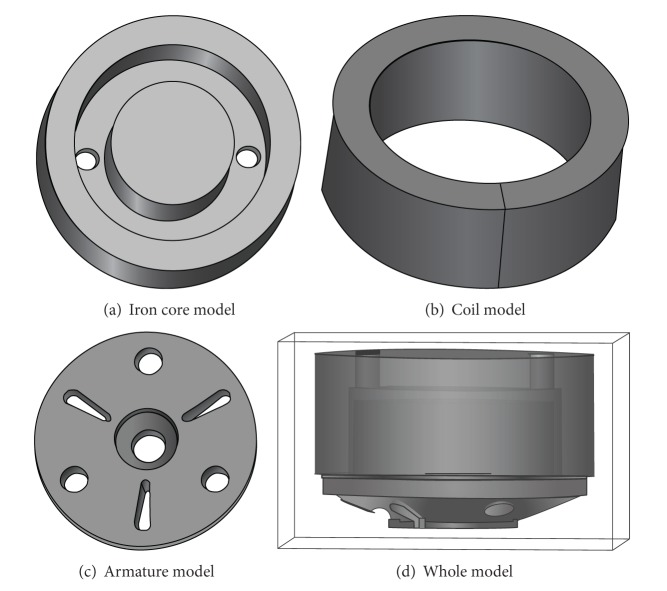
Ansoft simulation model of HSV.

**Figure 4:**
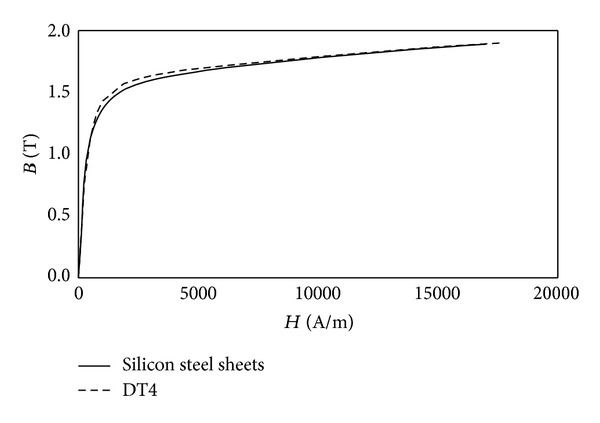
*B*-*H* curves of materials.

HSV is the nonaxisymmetrical structure therefore 3D model has been developed in Ansoft Maxwell environment to get the high precision results. Valve stem, armature reset spring, plug, spring seat, shell, and seal ring are nonmagnetic materials whose magnetic permeability are similar to air. Therefore, they have been ignored during modeling whereas iron core, coil, and armature have been considered. This cannot only improve computational efficiency, but also guarantee computational precision. Iron core model is shown in Figure 3(a). It is made of silicon steel sheets with lamination coefficient of 0.95. *B*-*H* curve of iron core material is shown in Figure 4. Coil model is shown in Figure 3(b). It is combination of a number of copper coils to build into a coil ring. Longitudinal section of the ring is made for the input terminals of excitation. The excitation type is set to current. DT4 (electrician pure iron) type armature model is shown in Figure 3(c). Finally infinite far field boundary condition has been established by using air surrounding the entire model as shown in Figure 3(d).

**Figure 5:**
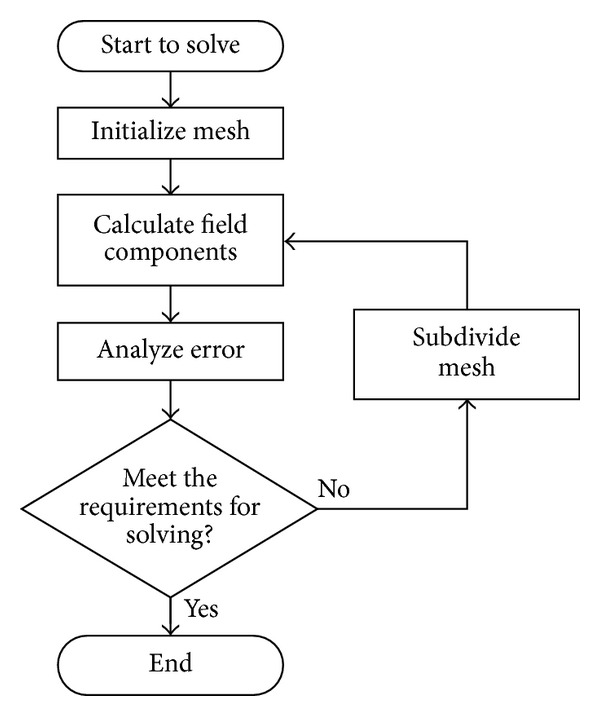
The method of adaptive mesh.

After developing the model and setting the axial electromagnetic force of armature as solution parameter, the model was solved by meshing in an adaptive method with gradual refining to meet energy error or achieve maximum iteration times.  Figure 5 shows the method of adaptive mesh.

### 2.2. Numerical Model Validation

**Figure 6:**
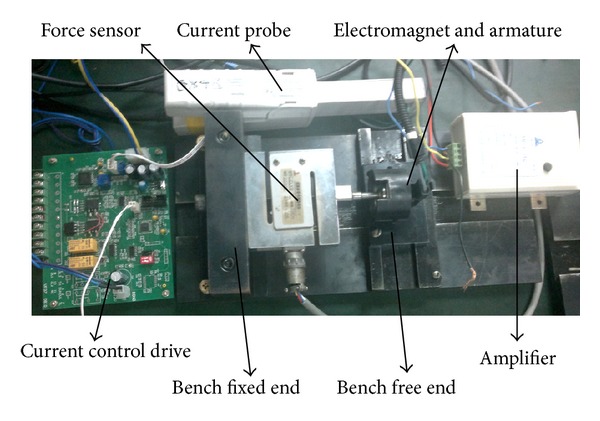
Test bench for HSV.

Amount of electromagnetic force driving the armature in the axial direction governs the driving capability of HSV. The electromagnetic force is measured in the test bench for HSV as shown in Figure 6.  Table 1 shows the measurement accuracy of main equipments.

Iron core is placed at the bench free end, while armature and force sensor of resistance strain connecting the armature are placed at the bench fixed end. Height of free end is adjusted such that the iron core axis and the armature axis are in the same horizontal line. Working air gap (the distance between iron core and armature) is changed by adjusting the distance between the free end and the fixed end. Armature is attracted towards the iron core after constant current to the coil is turned on, whereupon the force sensor of resistance strain generates a weak voltage signal. This voltage signal provides the size of electromagnetic force in the axial direction after passing through high precision amplifier. We have obtained multiple groups of experimental results by changing the working air gap and the size of constant current to the experimental setup.

**Figure 7:**
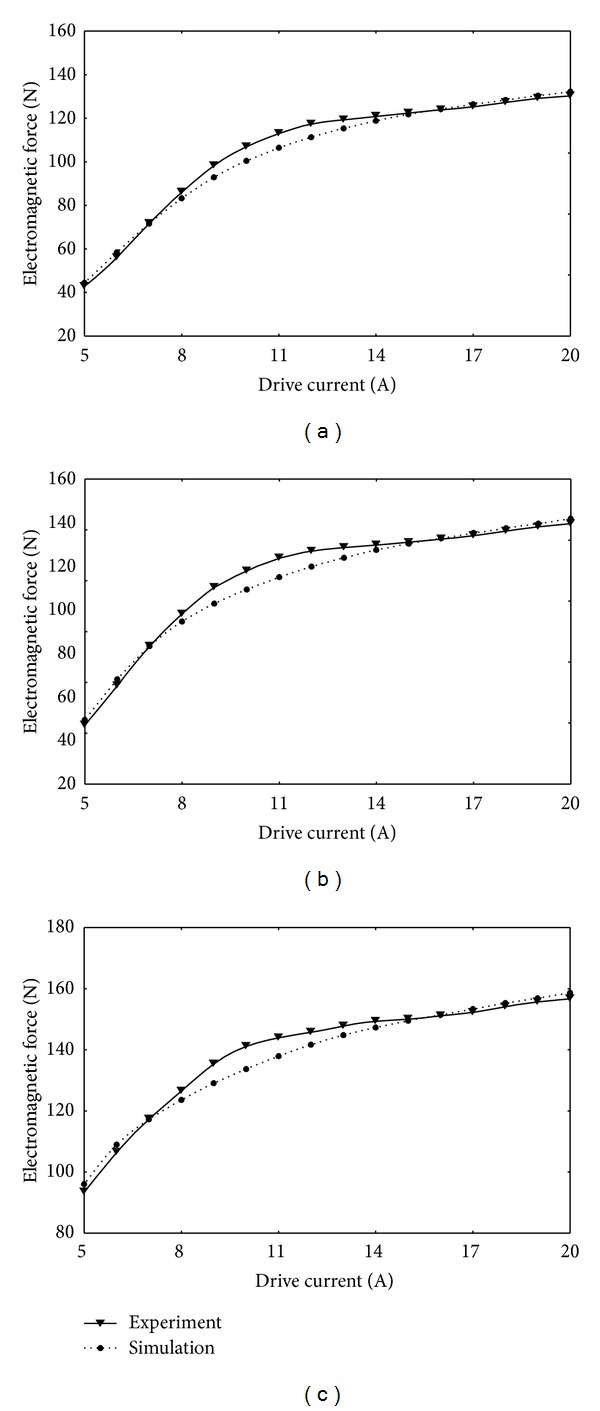
Comparison of experimental and simulated electromagnetic force at working air gaps of (a) 0.2 mm, (b) 0.15 mm, and (c) 0.1 mm.

Figures 7(a)–7(c) show comparisons between the simulated and experimental results of electromagnetic force at various drive currents and working air gaps. It is easy to infer that the simulated and experimental results match closely under the different drive currents with a maximum deviation of 6% which is within the acceptable range. Conclusively, our developed numerical model is coherent with experimental results and therefore can predict the electromagnetic force of solenoid valve quite accurately.

### 2.3. RSM

#### 2.3.1. RSM Theory

The RSM is first put forward by statisticians Box and Wilson in 1951 [15]. It comes from mathematical method, statistical analysis, and experimental design method and is often used to explore the mathematical model between the response output and influence factors of the unknown system or process [15]. The response surface model establishment for our research is as follows.

Let us assume that parameter or design point is the *n* dimensional vector (*x* ∈ *R* ^*n*^) and has the following relationship with the response *y*:

(5) $$\begin{array}{c} y\left(x\right)=f\left(x\right)+\varepsilon . \end{array}$$

However the true function relationship is unknown and can be very complex and *ε* is the error term. According to the engineering experience, usually using a second-order polynomial model replaces true function during a relatively small area. A general form of a second order polynomial model is as follows:

(6) y(x)=α0+∑i=1nαixi+∑i=1nαiixi2 +∑i<jnαijxixj+ε=∑i=0kαiφi(x)+ε.

In the above equation, *φ*_*i*_(*x*) is the basic function and *k* is the number of basic functions which equals (*n* + 1)(*n* + 2)/2.

Solve the unknown coefficient **A** = [*α*_0_,*α*_1_,…,*α*_*k*_]^*T*^ with the least square method:

(7) $$\begin{array}{c} E\left(\varepsilon \right)=\overset{q}{\underset{j=1}{\sum }}\varepsilon^{2}={\overset{q}{\underset{j=1}{\sum }}\left[y\left(x_{j}\right)-\overset{k}{\underset{i=0}{\sum }}\alpha_{i}\varphi_{i}\left(x_{j}\right)\right]}^{2},\\ {\frac{\partial E\left(\varepsilon \right)}{\partial \alpha_{i}}|}_{\alpha_{i}}=2X^{T}A-2X^{T}Y=0. \end{array}$$

From the above equations, we can get

(8) $$\begin{array}{c} A={\left(X^{T}X\right)}^{-1}X^{T}Y. \end{array}$$

In the above equation **Y** is the response vector at *q* experimental design points and **X** is the base function matrix. Experimental design points are obtained by the optimal Latin hypercube design (OLHD) and the response values are obtained by the numerical model of HSV.

#### 2.3.2. OLHD

Latin hypercube design (LHD) is one of space filler designs in which design space of each factor is evenly divided. These levels of factors are randomly combined to specify *n* points for defining the design matrix. The design points may distribute unevenly for high dimensional design space due to the random combination characteristics by LHD. But OLHD has better space filling and uniformity because it is based on LHD and applies a certain optimization algorithm to make the design points distribute as evenly as possible in the entire design space. Maximin distance criterion *φ*_*p*_ has been adopted in this paper [16].

#### 2.3.3. Response Surface Model Evaluation

Polynomial response surface model is usually evaluated by adj *R* ^2^ and *Q* ^2^. adj *R* ^2^ stands for the degree of consistency between the regression model and the experimental results for getting the regression model. *Q* ^2^ stands for the prediction ability of the regression model. Both values of adj *R* ^2^ and *Q* ^2^ are between 0 and 1. In general, values of adj *R* ^2^ and *Q* ^2^ are closer to 1, the model is considered better. Consider another case where adj *R* ^2^ is more than 0.9 and *Q* ^2^ is more than 0.5. Difference between adj *R* ^2^ and *Q* ^2^ is less than 0.3 which means that the model has a good consistency and prediction ability. If the difference is more than 0.3, the model is not very ideal [17]. Consider

(9) $$\begin{array}{c} \text{adj}\;R^{2}=1-\frac{{SS}_{\text{residual}}\left(df_{\text{residual}}+df_{\text{model}}\right)}{\left(SS_{\text{residual}}+SS_{\text{model}}\right)df_{\text{residual}}}, \end{array}$$

(10) $$\begin{array}{c} Q^{2}=1-\frac{\text{PRESS}}{SS_{\text{residual}}+SS_{\text{model}}}. \end{array}$$

In the above equations *SS*_residual_, *df*_residual_, *SS*_model_, *df*_model_, and PRESS are residual sum of squares, residual degree, regression sum of squares, regression degree and predicted residual sum of squares, respectively.

## 3. Result and Analysis

### 3.1. Single Parameter Analysis

**Figure 8:**
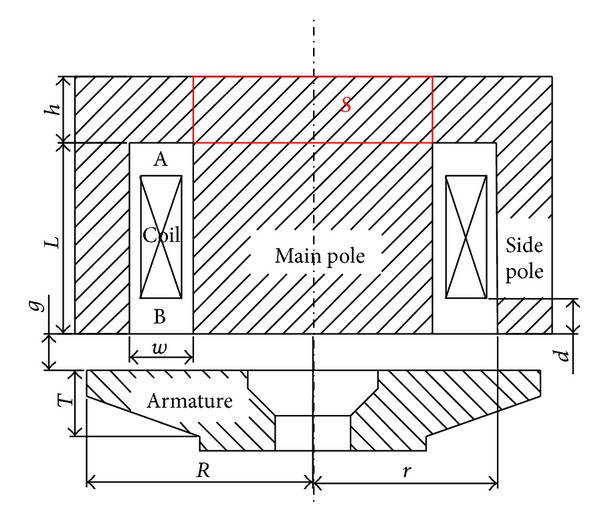
The schematic of electromagnet.

Parameters of HSV mainly include iron core, winding, armature, control, and assembly. This section presents an analysis of most of its parameters, some of which have been shown in Figure 8. All other parameters have been considered with their reference values when carrying out single parameter analysis.  Table 2 summarizes reference values and ranges of these parameters.

#### 3.1.1. Iron Core Parameters Analysis

**Figure 9:**
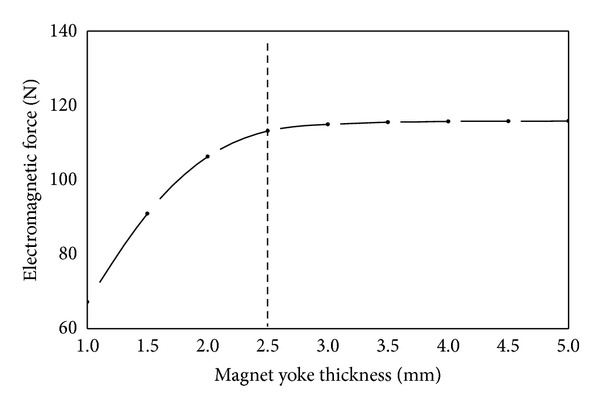
Influence of magnet yoke thickness on electromagnetic force.

(*1) Pole Length*. The maximum change of electromagnetic force is inconspicuous with a value of only 0.37 N when pole length changes from 6 to 9.2 mm. This behavior can be explained as follows. On one hand, the magnetic flux leakage in the region A (from coil upside to magnet yoke) as shown in Figure 8 increases with the increase of pole length, which decreases the magnetic resistance of the whole magnetic circuit. As a result, the electromagnetic force increases. On the other hand, the magnetic resistance of iron core increases with the increase of pole length, which decreases the electromagnetic force. However, the percentage of magnetic flux leakage is very small in the entire magnetic flux. Besides, the magnetic resistance of magnetic circuit mainly lies at the working air gap because the magnetic permeability of air is much less than that of the materials of iron core and armature. Therefore, the change of electromagnetic force caused by the pole length is slight. So the pole length just needs to be more than the coil height which can save the material and the volume of solenoid valve.

(*2) Magnet Yoke Thickness*. As shown in Figure 9, electromagnetic force increases rapidly with the increase of magnet yoke thickness from 1.0 mm to 2.5 mm, whereas the electromagnetic force increases inconspicuously when magnet yoke thickness is increased more than 2.5 mm. This behavior can be explained as follows. The electromagnetic force can be calculated by the following equation:

(11) $$\begin{array}{c} F=\frac{B^{2}S}{2\mu_{0}}. \end{array}$$

**Figure 10:**
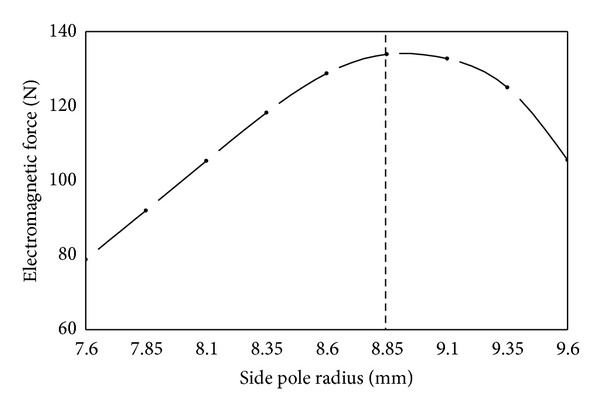
Influence of side pole radius on electromagnetic force.

In (11), *F* is in Newton (N) and stands for the electromagnetic force; *B* is in Tesla (T) and stands for the magnetic induction intensity of the air gap; *S* stands for the effective attracting area and is measured in m^2^; and *μ*_0_ stands for the magnetic permeability of vacuum with units of H/m. When magnet yoke thickness is small, the magnetic flux area of magnet yoke is also small which causes premature magnetic saturation. This restricts the increase of the main magnetic flux, which weakens the magnetic induction intensity of the air gap. With the increase of magnet yoke thickness, the magnetic saturation weakens, and the main magnetic flux increases rapidly. As a result, the magnetic induction intensity of the air gap becomes strong. Therefore, electromagnetic force increases rapidly. However, the magnetic flux area of magnet yoke is large enough when magnet yoke thickness is more than 2.5 mm which does not restrict the main magnetic flux. As a result, the magnetic induction intensity of the air gap does not change and therefore the change of electromagnetic force is not obvious any more. It has been found that the smallest magnetic flux area of the magnet yoke is close to the area of main pole. The smallest magnetic flux area of magnet yoke is approximately the lateral area “*S*” of cylinder as shown in Figure 8. Therefore, magnet yoke thickness can be half of main pole radius. It can ensure magnet yoke has enough area of magnetic flux to avoid getting magnetic saturation early. Besides, it also can save the material and the volume of solenoid valve.

(*3) Side Pole Radius*. As shown in Figure 10, side pole radius has an obvious effect on electromagnetic force. Electromagnetic force first increases and then decreases gradually with the increase of side pole radius. Reasons for this can be explained as follows. By keeping the coil width, namely, *w*, as shown in Figure 8 constant the side pole radius defines the effective main pole area. Therefore for smaller side pole radius main pole gets prematurely saturated which restricts the increase of the main magnetic flux. Similarly, with the increase of side pole radius the effective main pole area becomes large. As a result, the magnetic saturation weakens and electromagnetic force increases. However, when side pole radius is more than 8.85 mm the magnetic flux area of side pole is too small and as a result it gets magnetically saturated. The larger the side pole radius is beyond 8.85 mm, the worse the saturation and electromagnetic force will be. Therefore, when other parameters remain constant, the side pole radius has an optimal size.

#### 3.1.2. Coil Parameters Analysis

**Figure 11:**
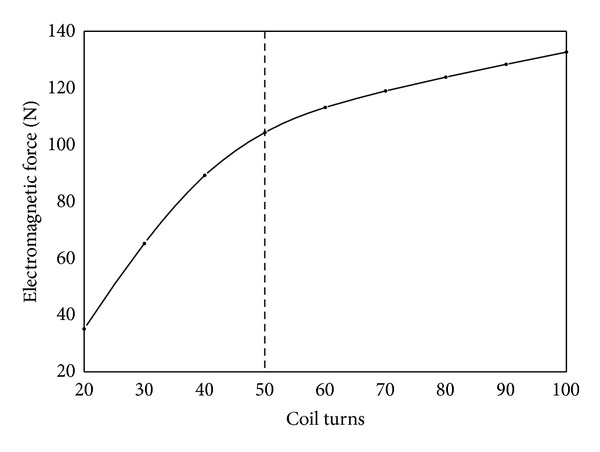
Influence of coil turns on electromagnetic force.

**Figure 12:**
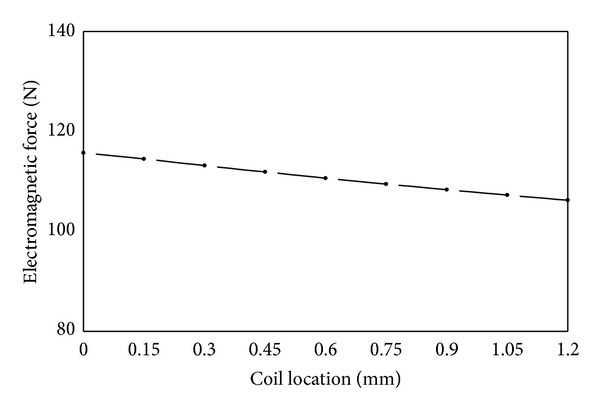
Influence of coil location on electromagnetic force.

(*1) Coil Turns*. As shown in Figure 11, electromagnetic force first increases rapidly and then slows down with the increase of coil turns. It can be explained as follows. Electromagnetic force is determined by the axial component of magnetic induction intensity at the working air gap. When drive current remains unchanged, the increase of coil turns increases magnetic potential. This leads magnetic induction intensity to increase at the working air gap, thereby increasing electromagnetic force. However when coil turns are more than 50, magnetic field gradually gets saturated. As a result, magnetic flux of magnetic circuit and magnetic induction intensity at the working air gap do not increase any more. Therefore the increase rate of electromagnetic force slows down. Any increase of coil turns will be a waste of material and will lead to the increase of the resistance of coil. This will cause the increase of loss and heat productivity of coil, which shortens the working life of solenoid valve. Moreover, it will also increase the inductance of coil which in turn will slow down the increase rate of drive current in the closing process of solenoid valve and decrease the rate of drive current in the opening process of solenoid valve. Therefore the increase of coil turns will result in worse dynamic response of HSV.

(*2) Coil Location*. As shown in Figure 12, electromagnetic force decreases slightly with the increase of distance of coil location from the working air gap. The change of coil location has an effect on the magnetic leakage flux of two spaces. One space is the region A, the other one is the region B (from coil downside to armature) as shown in Figure 8. The effect of magnetic leakage flux in the region A has been referred in the analysis of pole length before, it can decrease the magnetic resistance of the whole magnetic circuit. The magnetic leakage flux in the region B cannot flow through armature. This means it does not contribute to the increase of electromagnetic force and therefore it is useless. However when the coil is close to armature the magnetic leakage flux in the region A increases, and the magnetic leakage flux in the region B decreases. Thus it leads to stronger electromagnetic force. But the magnetic leakage flux is too small compared with working magnetic flux, so it has a limited impact for coil location on electromagnetic force. Coil is often sealed with phenolic resin to avoid any corrosion from fuel; therefore, there will always be a certain distance from coil to working air gap.

#### 3.1.3. Armature Parameters Analysis

**Figure 13:**
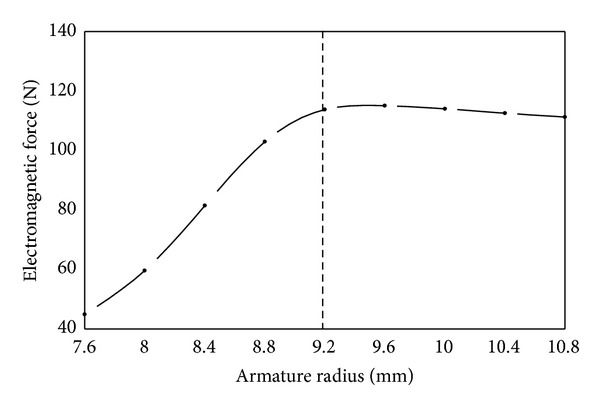
Influence of armature radius on electromagnetic force.

**Figure 14:**
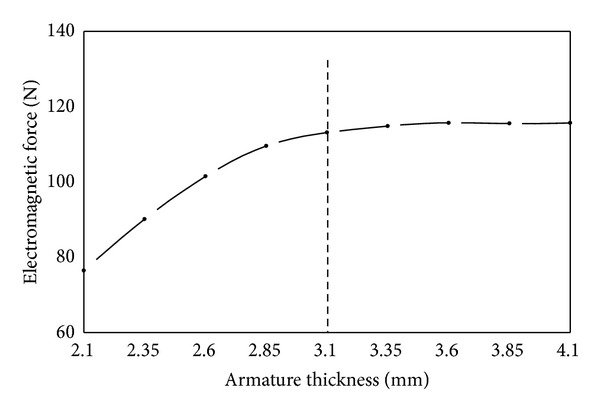
Influence of armature thickness on electromagnetic force.

(*1) Armature Radius*. As shown in Figure 13, electromagnetic force increases nearly linearly with the increase of armature radius from 7.6 mmm to 9.2 mm whereas it almost keeps unchanged with further increase of armature radius. The increase of armature radius increases the effective attracting area and consequently increased electromagnetic force. However, when armature radius is more than 9.2 mm, the effective attracting area almost remains unchanged. Therefore, the change of electromagnetic force is not obvious any more.

(*2) Armature Thickness*. As shown in Figure 14, the influence rule of armature thickness is similar to armature radius on the electromagnetic force. Electromagnetic force increases nearly linearly with the increase of armature thickness from 2.1 mm to 3.1 mm whereas it almost remains unchanged with further increase of armature thickness. One of the effects of increase of armature thickness is the decrease in the magnetic reluctance of the armature. This leads to increase of magnetic flux of working magnetic circuit and as a result electromagnetic force gets stronger. On the other hand, with the increase of armature thickness the magnetic flux area along the armature radial increases. As a result the magnetic induction intensity of armature decreases and it becomes difficult for the magnetic field to get prematurely saturated in armature. However, the magnetic flux area of armature is large enough when magnet yoke thickness is more than 3.1 mm and armature thickness does not have obvious influence on magnetic field in this situation. Therefore, the change of electromagnetic force is not evident any more as shown in Figure 14.

#### 3.1.4. Assembly Parameters Analysis

**Figure 15:**
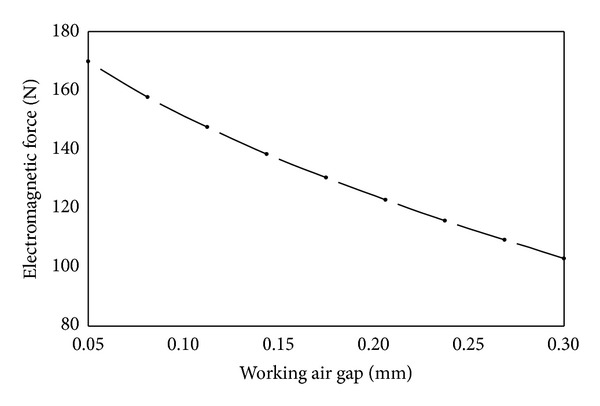
Influence of working air gap on electromagnetic force.

(*1) Working Air Gap*. As shown in Figure 15, electromagnetic force decreases approximately linearly with the increase of working air gap. The magnetic permeability of air is much less than that of the materials of iron core and armature. Therefore the total magnetic reluctance is mainly concentrated on the place of working air gap. Besides, magnetic reluctance is proportional to width of air gap. Therefore with the increase of working air gap the magnetic reluctance increases and the electromagnetic force decreases.

#### 3.1.5. Control Parameters Analysis

**Figure 16:**
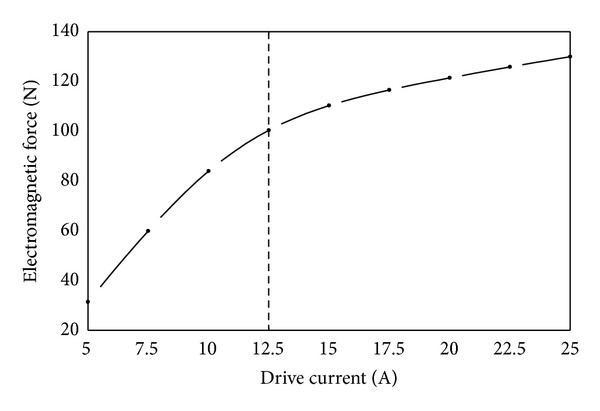
Influence of drive current on electromagnetic force.

(*1) Drive Current*. As shown in Figure 16, the influence rule of drive current is similar to coil turns on the electromagnetic force. Electromagnetic force first increases rapidly then increases slowly with the increase of drive current. It can be explained as follows. When coil turns remain unchanged, the increase of drive current increases the magnetic potential. This increases the magnetic induction intensity at the working air gap, thereby increasing electromagnetic force. However when drive current is more than 12.5 A, magnetic field gradually gets saturated. Magnetic flux of magnetic circuit and magnetic induction intensity at the working air gap does not increase any more. Therefore the increase rate of electromagnetic force slows down. Besides, the bigger the drive current is, the more the loss and heat productivity of coil are. Increases in drive current will shorten the working life of solenoid valve. Therefore we can assert that the drive current should be just enough to meet the working requirements.

### 3.2. Two Parameters Interaction Effect Analysis

Through single parameter analysis we can determine the appropriate pole length, coil location, and magnet yoke thickness. Because pole length and coil location do not have obvious influence on the electromagnetic force therefore pole length is set for 1 mm greater than the distance from the bottom of the coil. The space is used for coil skeleton and seal. The coil location is determined by keeping 0.3 mm between coil and working air gap (*d* = 0.3 mm, actual sealing thickness). In addition, magnet yoke thickness is given half the magnet pole radius. Therefore the interaction effect has been analyzed between remaining six parameters only.

*k* is 28 for six parameters system. According to the scale and complexity of the system the number of design points should increase properly. It is proposed by Sacks et al. [18] that for 5~10 parameters system, the number is 1.5*k* and for 20~30 parameters system, the number is 4.5*k*. In this paper the numbers 1.5*k*, 2*k*, and 2.5*k* of design points are designed, respectively, by the OLHD, and their response values are calculated by numerical simulation. Unknown coefficients of (6) are obtained by the least squares method.  Table 3 summarizes the model precision of different number design points. We can assert from Table 3 that numerical model with number of design points 2.5*k* has the highest precision and therefore its coefficients and significant values are given in Table 4.

If the coefficient *P* of the item is more than 0.1, it is regarded as insignificant factor. Therefore, this section will only present an analysis of the interaction between significant factors whereas analysis of insignificant factors will be ignored. For example interaction between working air gap and armature radius, armature thickness and armature radius, drive current and armature thickness, coil turns and side pole radius, armature thickness and side pole radius, and finally armature radius and side pole radius are significant factors and will be discussed in what follows.

Analysis is limited to the interaction between two parameters only; therefore, the values of other parameters have been assigned their appropriate intermediate values. Corresponding electromagnetic force contour map has also been constructed and analyzed.

#### 3.2.1. Interaction between Working Air Gap and Armature Radius

**Figure 17:**
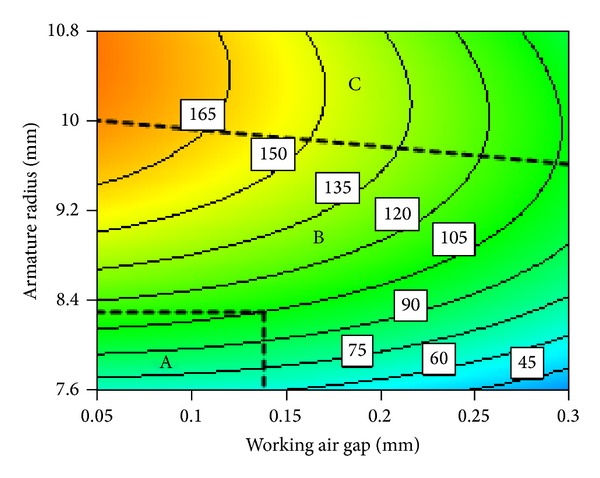
Interaction of working air gap and armature radius.

Figure 17 shows electromagnetic force contour map of interaction between working air gap and armature radius. After analyzing Figure 17, we can divide interaction between working air gap and armature radius into almost three regions of relations, namely, A, B, and C. In the region A, electromagnetic force contour map is almost horizontal and armature radius nearly does not change with the increase of the working air gap. In the region B, while electromagnetic force remains constant, an approximate linear relationship can be observed between working air gap and armature radius. In the region C, electromagnetic force contour map is almost vertical, while working air gap almost does not change with the increase of armature radius.

**Figure 18:**
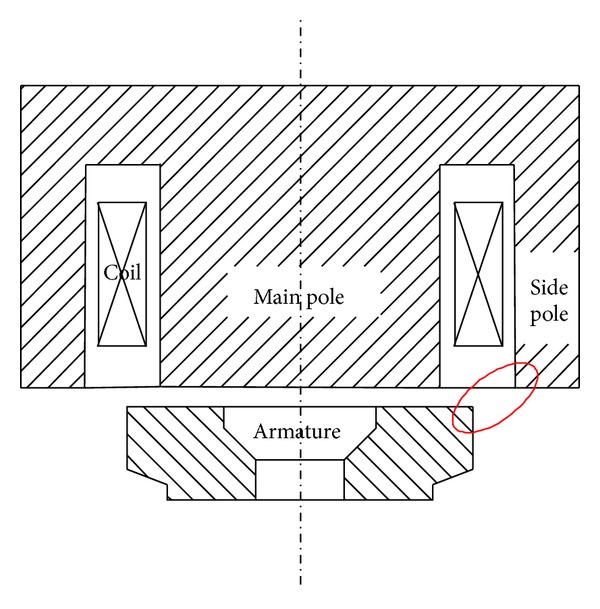
Size relationship between armature radius and side pole radius.

Area wise relations can be explained as follows. Region A is where both working air gap and armature radius are relatively small; armature radius is less than side pole radius as shown in Figure 18. Due to lesser attracting area between armature and side pole, magnetic lines gather in the red region, as a result magnetic saturation takes time in this place. Working air gap is not sensitive to the change of armature radius in the region A. Therefore contour map is nearly a horizontal line as shown in the region A. With the further increase of working air gap, magnetic reluctance increases much, and then magnetic saturation disappears. This leads to decrease of magnetic induction intensity as a result electromagnetic force decreases. However, with the increase of armature radius, the effective attracting area increases. This results in increase of electromagnetic force again. This is why we get linear increase in region B. When armature radius increases to a certain value, the effective attracting area almost does not change any more. Therefore armature radius does not have obvious influence on working air gap. Therefore, the contour map is almost vertical line as shown in region C of Figure 17.

#### 3.2.2. Interaction between Drive Current and Armature Thickness

**Figure 19:**
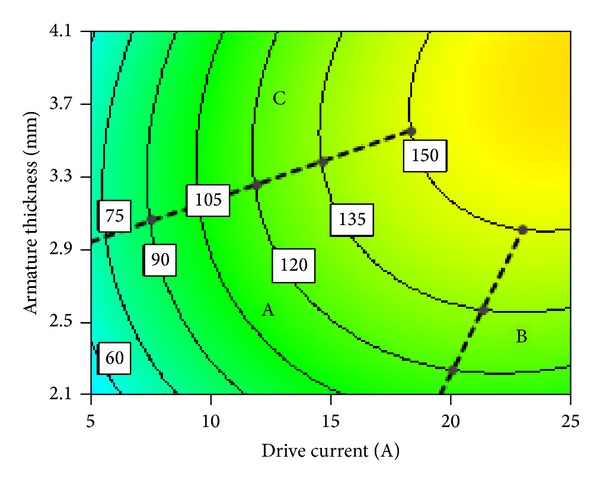
Interaction of drive current and armature thickness.

Figure 19 shows electromagnetic force contour map of interaction between drive current and armature thickness. We can also divide interaction of drive current and armature thickness into almost three regions of relations, namely, A, B, and C, as shown in Figure 19. In the region A, a negative correlation can be observed between drive current and armature thickness; that is, one increases, while the other decreases. In the region B, contour lines of electromagnetic force are approximate horizontal lines, which means drive current does not have obvious influence on armature thickness. In the region C, contour lines of electromagnetic force are approximate vertical lines, which means armature thickness does not have obvious influence on drive current.

These relations can be explained as follows. Region A is where both drive current and armature thickness are relatively small. Electromagnetic force increases with the increase of either drive current or armature thickness in this region. Therefore, drive current and armature thickness are negatively correlated in this region. However, when armature thickness is constant, its radial effective area of magnetic flux is also constant. Besides the increase of drive current will lead to the increase of magnetic flux. Therefore as drive current increases to a certain value, armature gets magnetically saturated. Thus electromagnetic force will not change obviously with the increase of drive current. Therefore contour map is nearly a horizontal line as shown in the region B. Similarly, when armature thickness increases to a certain value, its radial effective area of magnetic flux will become large enough. Armature thickness does not restrict the increase of magnetic flux any more in the situation. However, drive current and magnetic potential are constant. Magnetic flux of magnetic circuit almost remains unchanged and therefore electromagnetic force will not change obviously with the increase of armature thickness. Contour map in this condition is nearly a vertical line as shown in the region C. At last, we can find that both saturated drive current and armature thickness (black symbols) increase with the increase of electromagnetic force. It is suggested that drive current and armature thickness should be selected in the region A. Selection of these parameters in region A can have two benefits. Firstly, it can lead to select light moving parts of HSV resulting in improved dynamic response speed of HSV. Secondly, it can help in selecting low input power and minimizing the loss and heat production of coil.

#### 3.2.3. Interaction between Coil Turns and Side Pole Radius

**Figure 20:**
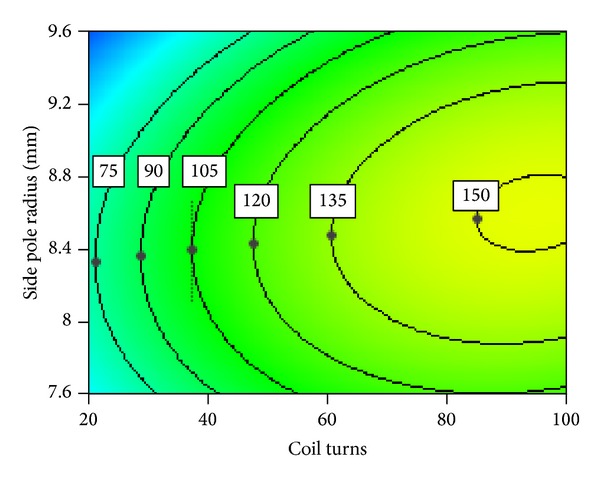
Interaction of coil turns and side pole radius.

A series of inflection points (black symbols) are shown in Figure 20 representing the electromagnetic force contour map of interaction between coil turns and side pole radius. A negative correlation can be observed between coil turns and side pole radius where their values are lower than inflection points. This means that coil turns increase despite of the decrease of side pole radius. A positive correlation can be observed between coil turns and side pole radius, where their values are more than inflection points. This means that coil turns increase with the increase of side pole radius. Moreover electromagnetic force contour map curves above and below the inflection points become more flat with the increase of electromagnetic force. Because the stronger electromagnetic force is, the more coil turns is and the closer magnetic field is to saturation, so the side pole radius is not more sensitive to the change of coil turns. As a result the curves become more flat. It is suggested that coil turns and side pole radius should be selected at the inflection points so that minimum coil turns can be selected for a given electromagnetic force.

#### 3.2.4. Interaction between Armature Thickness and Armature Radius

**Figure 21:**
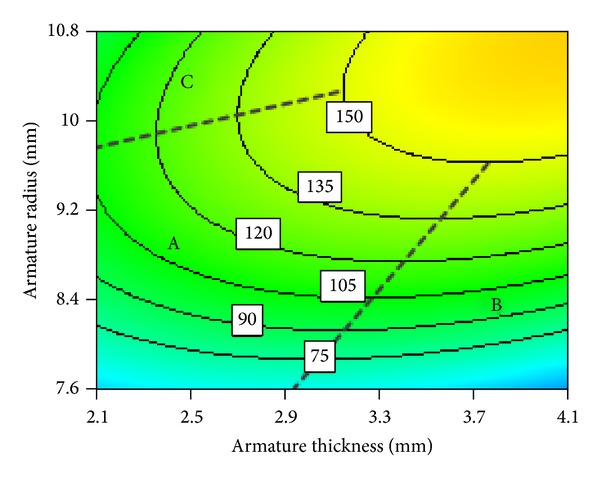
Interaction of armature thickness and armature radius.

Figure 21 shows electromagnetic force contour map of interaction between armature thickness and armature radius. We can also divide this interaction relation into almost three regions of relations, namely, A, B, and C, as shown in Figure 21. In the region A, approximately negative linear relationship is observed between the two parameters. In the regions B and C contour lines of electromagnetic force are approximately horizontal and vertical, respectively. This shows that interaction between the two parameters is weak. These relations can be explained as follows. Region A is where both armature thickness and armature radius are relatively small. Electromagnetic force increases with the increase of armature thickness and armature radius. Therefore, the relationship between armature thickness and armature radius is approximately negative linear in this region. However, in the regions B and C armature thickness and armature radius are relatively big. In these conditions armature's radial effective area of magnetic flux will be large enough and its effective attracting area almost does not change any more. Therefore they do not have obvious influence on the electromagnetic force as a result the interaction between these two parameters become weak. It is suggested that armature thickness and armature radius should be selected in the region A so that their values can be as small as possible. This will make mass of moving parts of HSV light improve the dynamic response speed of HSV.

#### 3.2.5. Interaction between Armature Thickness and Side Pole Radius

**Figure 22:**
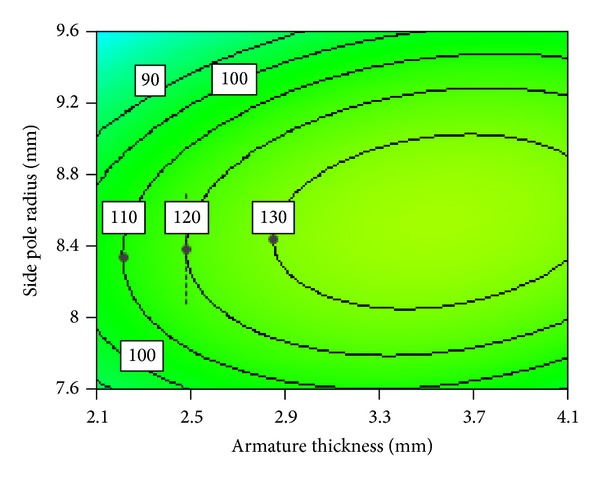
Interaction of armature thickness and side pole radius.

As shown in Figure 22, interaction between armature thickness and side pole radius is similar to interaction between coil turns and side pole radius. A series of inflection points (black symbols) exist in the electromagnetic force contour map. A negative correlation can be observed between armature thickness and side pole radius within certain realms, where they are under an inflection point. A positive correlation can be also observed between armature thickness and side pole radius within certain realms where they are above an inflection point. Moreover as armature thickness increases to a certain value, the electromagnetic force contour lines becomes flat. This shows that the influence of armature thickness on the side pole radius gradually weakens. As mentioned before, when armature thickness increases to a certain value, its radial effective area of magnetic flux will become large enough. Therefore armature thickness does not restrict the increase of magnetic flux any more in the situation. As a result electromagnetic force will not change obviously with the increase of armature thickness and therefore the influence of armature thickness on the side pole radius gradually weakens. It is also suggested that armature thickness and side pole radius should be selected at the inflection point so that the minimum value of armature thickness can be selected with given electromagnetic force. This will make mass of moving parts light and help in improving the dynamic response speed of HSV.

#### 3.2.6. Interaction between Armature Radius and Side Pole Radius

**Figure 23:**
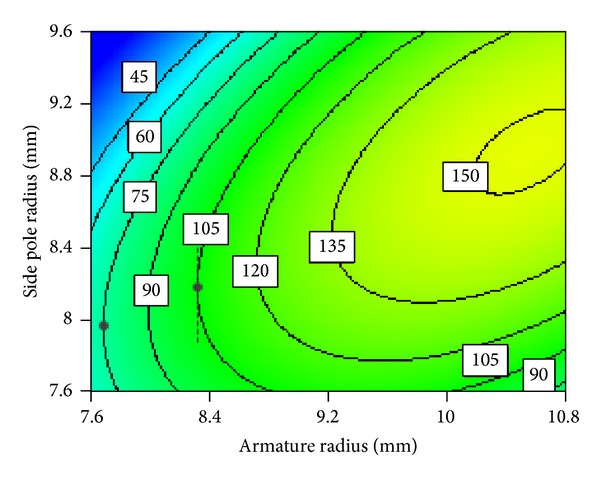
Interaction of armature radius and side pole radius.

A series of inflection points (black symbols) also exist in the electromagnetic force contour map between armature radius and side pole radius as shown in Figure 23. Below the inflection points, the side pole radius decreases first and then increases gradually with the increase of armature radius, while above the inflection points the armature radius increases gradually with the increase of the side pole radius. This behavior can be explained as follows. There is an optimal value of side pole radius matching an armature radius, which makes the electromagnetic force the biggest. This value is at the inflection point. On one hand, when the side pole radius is less than the inflection point, electromagnetic force increases with the increase of the side pole radius. On the other hand, when the side pole radius is more than the inflection point, electromagnetic force decreases despite of the increase of the side pole radius. Besides, electromagnetic force first increases then slightly decreases with the increase of armature radius as shown in Figure 23. It is suggested that armature radius and side pole radius should be selected at the inflection point so that the armature radius can be minimum with the given electromagnetic force. This helps in selecting lighter moving parts and improving the dynamic response speed of HSV.

## 4. Conclusion

A numerical model of HSV has been developed in Ansoft Maxwell environment with a good accuracy. It provides an effective platform for the research on the electromagnetic force characteristic of HSV for EUP.

The influence rules of various parameters on the electromagnetic force have been obtained. It has been found out that pole length and coil location do not have obvious influence on the electromagnetic force whereas other parameters like magnet yoke thickness, side pole radius, coil turns, armature radius, armature thickness, and working air gap have a great influence on electromagnetic force. It is suggested that pole length should be as small as possible; coil should be as close to the working air gap as possible; and magnet yoke thickness should be half of main pole radius.

It is concluded that interaction factors such as working air gap with armature radius; drive current with armature thickness; coil turns with side pole radius; armature thickness with its radius; armature thickness with side pole radius; and armature radius with side pole radius have significant influences on the electromagnetic force. It has been found that a best match value exists between coil turns and side pole radius; armature thickness and side pole radius; and armature radius and side pole radius.

## Figures and Tables

**Table 1:** Measurement accuracy of main equipments.

Equipment	Force sensor	Current probe
Type	CZLYB-3	1146A
Producer	Chengdu Xingpu transducer Co., Ltd.	Agilent technology
Measurement range	0~700 N	1~100 A
Measurement accuracy	≤±0.05%	≤±4%

**Table 2:** Important parameters of model.

Parameters	Reference value	Range
Iron core		
Pole length *L* (mm)	6.8	6~9.2
Magnet yoke thickness *h* (mm)	2.5	1~5
Side pole radius *r* (mm)	8.25	7.6~9.6
Coil		
Turns (N)	60	20~100
Location *d* (mm)	0.3	0~1.2
Armature		
Radius *R* (mm)	10.2	7.6~10.8
Thickness *T* (mm)	3.1	2.1~4.1
Assembly		
Working air gap *g* (mm)	0.25	0.05~0.3
Control		
Drive current *I* (A)	16	5~25

**Table 3:** The precision of different model.

	1.5*k*	2*k*	2.5*k*
adj *R* ^2^	0.9185	0.9150	0.9219
*Q* ^2^	0.7535	0.8013	0.8634

**Table 4:** The coefficients and their significant values of model.

Equation item	Constant	*g*	*I*	*N*	*T*	*R*	*r*
Coefficient	−1410.4	456.44	2.9128	−0.40302	−105.29	44.343	301.70
*P*		<0.01	<0.01	<0.01	<0.01	<0.01	0.01

Equation item	*g*∗*I*	*g*∗*N*	*g*∗*T*	*g*∗*R*	*g*∗*r*	*I*∗*N*	*I*∗*T*

Coefficient	−0.39518	0.31047	−42.872	−71.345	27.465	0.0024183	1.0720
*P*	0.93	0.78	0.32	0.02	0.53	0.86	0.06

Equation item	*I*∗*R*	*I*∗*r*	*N*∗*T*	*N*∗*R*	*N*∗*r*	*T*∗*R*	*T*∗*r*

Coefficient	0.048570	0.47869	0.16838	0.061084	0.22984	13.309	9.1458
*P*	0.89	0.38	0.22	0.48	0.10	<0.01	0.10

Equation item	*R*∗*r*	*g* ^2^	*I* ^2^	*N* ^2^	*T* ^2^	*R* ^2^	*r* ^2^

Coefficient	22.258	−456.48	−0.22622	−0.014508	−16.078	−13.104	−33.051
*P*	<0.01	0.37	<0.01	<0.01	0.05	<0.01	<0.01
